# Epstein–Barr Virus: Diseases Linked to Infection and Transformation

**DOI:** 10.3389/fmicb.2016.01602

**Published:** 2016-10-25

**Authors:** Hem C. Jha, Yonggang Pei, Erle S. Robertson

**Affiliations:** Department of Otorhinolaryngology–Head and Neck Surgery and Tumor Virology Program, Abramson Cancer Center, Perelman School of Medicine at the University of Pennsylvania, PhiladelphiaPA, USA

**Keywords:** EBV, latency, lytic, transformation, B-cell, epithelial cell, cancer, infection

## Abstract

Epstein–Barr virus (EBV) was first discovered in 1964, and was the first known human tumor virus now shown to be associated with a vast number of human diseases. Numerous studies have been conducted to understand infection, propagation, and transformation in various cell types linked to human diseases. However, a comprehensive lens through which virus infection, reactivation and transformation of infected host cells can be visualized is yet to be formally established and will need much further investigation. Several human cell types infected by EBV have been linked to associated diseases. However, whether these are a direct result of EBV infection or indirectly due to contributions by additional infectious agents will need to be fully investigated. Therefore, a thorough examination of infection, reactivation, and cell transformation induced by EBV will provide a more detailed view of its contributions that drive pathogenesis. This undoubtedly expand our knowledge of the biology of EBV infection and the signaling activities of targeted cellular factors dysregulated on infection. Furthermore, these insights may lead to identification of therapeutic targets and agents for clinical interventions. Here, we review the spectrum of EBV-associated diseases, the role of the encoded latent antigens, and the switch to latency or lytic replication which occurs in EBV infected cells. Furthermore, we describe the cellular processes and critical factors which contribute to cell transformation. We also describe the fate of B-cells and epithelial cells after EBV infection and the expected consequences which contribute to establishment of viral-associated pathologies.

## Introduction

### Viral Infections in Cancer

Hem C. Jha, Centre for Biosciences and Biomedical Engineering, Indian Institute of Technology Indore, Indore, India

Worldwide cancer is one of the leading causes of death (de Martel et al., 2012). In 1990, studies suggested that approximately 16% of all cancers are associated with infectious agents (Pisani et al., 1997). However, less developed regions range greater than 20% compared to the more developed regions which shows that approximately 9% of all cancers in the population are associated with infectious agents (de Martel et al., 2012). Interestingly, reports from 2002 estimates suggest that infectious agents are linked to 18% of the global burden of cancer (Parkin, 2006). In the same year, the share of infection associated cancers in less developed regions were more than 26% compared to 8% in more developed regions (Parkin, 2006). Further a study in 2008 suggested that these infection-associated cancers attributed to 33% of cancers in sub-Saharan Africa (de Martel et al., 2012). This study also showed that 30% of cancers associated with infections occurred in people younger than 50 years of age (de Martel et al., 2012), and strongly suggested that they are causative or major drivers in the process. In 2010, approximately 8 million deaths were linked to cancer. Surprisingly, and worrisome the rate of death due to cancer has continued to increase by 2% per year (Lozano et al., 2012). Thus, assessing the risk factors or causative agents of cancer is of extreme importance for future prevention strategies.

### EBV Types and Infection

Epstein–Barr virus (EBV) was first discovered in cultured tumor cells derived from a biopsy of a Burkitt’s lymphoma (BL) patient in 1964 (Burkitt, 1969). Alone, EBV accounts for 0.5–2% cancers based on geographical regions (Epstein et al., 1964; Khan and Hashim, 2014), and recent analysis indicates that 1.8% of all cancer deaths in 2010 were associated with EBV (Khan and Hashim, 2014). EBV was identified as the first human tumor virus as it was involved initially linked to BL, and since then several other types of cancers (Thompson and Kurzrock, 2004). There are two different EBV types which are commonly known as types 1 and 2 (Cho et al., 1999). It has been shown that the genomes of these two EBV types are very similar except for regions of the EBNA genes (Dambaugh et al., 1984). Tzellos et al. (2014) demonstrated that the greater ability of type 1 EBV infection to induce B cell proliferation is likely due to superior expression of EBNA2 which consequently leads to the upregulation of LMP1 and cellular CXCR7. Notably, the percent association of EBV varies in different diseases and may have consequences toward the aggressivity of the associated cancers.

## EBV Associated Diseases

### B-Cell Associated Diseases

#### Burkitt’s Lymphoma

Geographic regions like equatorial Africa and Papua New Guinea have holoendemic malaria and those are 100% EBV positive (Kelly and Rickinson, 2007). This is classified as endemic BL as typically represents as a jaw or abdominal tumor in children’s (Valenzuela-Salas et al., 2010). However, western countries have lower incidence rates and lower association with EBV. Approximately, 15–20% BL tumors were EBV positive (Kelly and Rickinson, 2007). Hence, it is possible that chronic immune stimulation from the burden of pathogenic agents may increase the incidence of BL (Engels, 2007). Interestingly, AIDS-BL, was shown to be very common among HIV-infected individuals with a wide range of CD4 counts, and usually appears as one of the symptoms of AIDS with 30–40% of these BL-tumors are positive for EBV. Typically, the majority of BLs carry a reciprocal translocation that places the c-myc gene under the control of either the heavy- or light-chain immunoglobulin (IgH or IgL) loci (Brady et al., 2007). Therefore, c-myc deregulation represents an essential defining feature of BL pathogenesis (Kelly and Rickinson, 2007). Interestingly, EBV genomes are found in neoplastic cells of endemic Burkitt’s lymphoma (eBL) patients (Wright, 1971; Tao et al., 1998). Endemic BL has been associated with endemic malaria (de-The, 1985). However, further studies are needed to effectively determine the contributions of malaria infection to development of EBV associated endemic BLs.

#### Post-transplant Lymphoproliferative Disease (PTLD) and Central Nervous System Lymphoma (CNS Lymphoma)

More than 90% of B-cells in early onset and 60–80% in late onset of post-transplant lymphoproliferative disease (PTLD) are EBV positive (Petrara et al., 2015). Moreover, early onset PTLDs are either polyclonal or oligoclonal, while most of late onset PTLDs are truly monoclonal (Carbone et al., 2008). PTLD occurs after transplantation or primary EBV infection, acquired from the donor following transplantation (Allen et al., 2002). As all the major EBV latent antigens are extensively expressed in PTLD, this strengthens the contributory role for EBV infection and viral gene expression in PTLD pathogenesis (Odumade et al., 2011). Interestingly, 50% of PTLD’s are deficient in a functional B-cell receptor (BCR), which is essential for B-cell survival (Bechtel et al., 2005). Therefore, EBV can protect these cells from death by blocking apoptosis even in the absence of antigen stimulation (Spender and Inman, 2011).

MicroRNAs refer to a group of small non-coding RNA molecules with crucial influences on specific cancers (Macfarlane and Murphy, 2010). By detecting the EBV and cellular microRNAome in PTLD (Forte et al., 2012; Harris-Arnold et al., 2015), studies showed that there are two different microRNA profiles identified in primary central nervous system post-transplant lymphoproliferative disorders (pCNS PTLD). First, EBV microRNAs interacts with the cellular microRNAome similarly to that of EBV-associated systemic PTLD and the second could be limited to the immunological functions associated with the central nervous system (Moscato et al., 2013; Fink et al., 2014). Although, a limited expression of latency genes is also seen in EBV associated systemic PTLD, based on promoter utilization it is still considered to be latency III (Fink et al., 2014). A higher frequency of EBV in pCNS PTLD compared to systematic PTLD may result in pathological differences (Cavaliere et al., 2010). Also, AIDS-related CNS lymphomas are derived from germinal center B-cells and are always EBV positive (Bibas and Antinori, 2009). These CNS lymphomas contain immunoblastic and large non-cleaved lymphomas (Taylor et al., 1978). EBV infection in PTLD exploits several strategies to ensure persistent infection, namely, prevention from death of infected cells, enhancement of their proliferation to maintain the infected reservoir, and escape from host immune system (Hsieh et al., 1999; Tanner and Alfieri, 2001).

#### Hodgkin Lymphoma (HD)

In 1966, MacMahon suggested that Hodgkin’s disease might be due to an infectious agent (Flavell and Murray, 2000). Later, the infectious agent EBV has been detected by high antibody titers in patients with Hodgkin’s disease when compared with other lymphomas patients (Alexander et al., 2003). EBV positivity in HD is extremely high in some geographical areas (Flavell and Murray, 2000). There are several critical factors on which association of EBV with Hodgkin’s disease seems to depend, which include geography, histological subtype, sex, ethnicity, and age (Flavell and Murray, 2000). Reports have suggested that in less developed regions, 90% of childhood Hodgkin lymphoma (HD) and approximately 60% of adult HDs are EBV positive (Oh and Weiderpass, 2014). Earlier Weniger and Kuppers (2016), reviewed that LMP1 and LMP2A are expressed at high levels in the Reed-Sternberg cells, and activate pathways, namely NF-kB and PI3K which are also highly activated in EBV-negative HL. However, the role that EBV plays in their pathogenesis is still not fully understood. Contrary to this, gastrointestinal involvement is an exceptionally rare incident in HD and might occur as infiltration from mesenteric lymph nodes (Issa and Baydoun, 2009). Few cases have been described that demonstrate EBV positive primary gastrointestinal HD (Cardona et al., 2012). Initial symptoms are restricted to extranodal tissue and are more uncommon in HD compared to NHLs. Few cases have been described which demonstrate EBV positive primary gastrointestinal HD (Cardona et al., 2012). The majority of these cases were linked to Crohn‘s disease and immunosuppression (Lewis, 2012). A probable hypothesis given for EBV infected HL in children and older individuals was primary infection and a weak immune response toward EBV latent genes, respectively (Grywalska and Rolinski, 2015). Furthermore, delayed primary infection can contribute to EBV associated HL in young adults (Grywalska and Rolinski, 2015).

#### Non-Hodgkin Lymphoma (NHL)

Eighty five to ninety percentage of Non-Hodgkin’s lymphomas (NHLs) arises from B lymphocytes followed by T lymphocytes and natural killer (NK) lymphocytes (Diaz et al., 1993). Lymph nodes are the primary site for NHL, however, it can occur in almost any tissue (Krol et al., 2003). NHL attributed approximately 5.1% of all cancer cases and 2.7% of all cancer deaths worldwide (Sharma et al., 2014). Geographically, NHL presence is predominantly observed in North America, Europe, Oceania, and several African countries (Hossain et al., 2014). There are several risk factors for NHL including sex, age, HIV/AIDS, familial aggregation, autoimmune conditions, rheumatoid arthritis, celiac disease, systemic lupus erythematosus (Ekstrom Smedby et al., 2008). There are also several microbial agents shown to be associated with NHL with one of the prominent one being EBV (Carbone et al., 2008).

#### EBV-Associated Lymphomas in Congenital Immunodeficient Individuals

First, these disorders are a result of an inherited immunodeficiency known as X-linked lymphoproliferative disorder (Marsh and Filipovich, 2011). Second, some lymphomas are related to use of immunosuppressive drugs given to transplant recipients. Third, they are due to immunosuppression from HIV infection and referred to as AIDS-related lymphoproliferative disorders (Kanakry and Ambinder, 2013). Typically the gene expression patterns in these disorders show EBV latency III (Thompson and Kurzrock, 2004). Furthermore, EBV-associated lymphomas in the immunocompromised hosts are very aggressive in nature and extremely difficult to treat (Cohen et al., 2009).

#### Oral Hairy Leukoplakia

Oral hairy leukoplakia (OHL) presents as white patches, which are observed normally on the lateral surfaces of the tongue (Komatsu et al., 2005). The presence of EBV is detected in the tissues and blood due to chronic immunosuppression like HIV infection (De Paschale and Clerici, 2012). Additionally, patients who receive organ and bone marrow transplant have also been shown to develop these symptoms (Allen et al., 2002). Therefore ORL correlates with ongoing immune suppression due to HIV infection or treatment post-transplantation to prevent rejection.

### Epithelial Cells Related Cancers

#### Nasopharyngeal Carcinoma (NPC)

Nasopharyngeal cancer (NPC) is an invasive malignancy rarely found in western countries, however, high prevalence is observed in populations like, South-Eastern Asia and Northern Africa, mainly in Southern China, Singapore, Malaysia, and North-Eastern India (Torre et al., 2015). The majority of NPC tumors are found to be positive for EBV infection (Niedobitek, 2000). Establishment of a latent and transforming infection in epithelial cells are potentially an important causative factor for the development of NPC (Tsao et al., 2015). Studies suggest that about two thirds of NPC cases present as type II EBV latency, in which viral latency antigens are less immunogenic, but could still be targeted by specific cytotoxic T-lymphocytes (Rooney et al., 2014). Predominantly, EBNA1 and LMP1 are expressed in the majority of EBV-positive NPCs (Brooks et al., 1992).

There are three types of NPC as classified by World Health Organization (WHO). Type one is characterized as keratinizing squamous cell carcinoma (SCC), type two is non-keratinizing carcinoma, and type three is undifferentiated carcinoma (Pathmanathan et al., 1995). Usually, types 1 and 2 have been associated with western population (Cao et al., 2011). Type three which is predominantly associated with EBV latent infection have been associated with China and some other Asian countries (Cao et al., 2011). Due to unusual lymphocytic infiltration, type three NPC is referred as lymphoepithelioma of the nasopharynx, and reports have suggested that EBV particles through cell–cell contact can be transferred from lymphoid cells to nasopharynglial epithelial cells (Chang et al., 1999). A very high-grade dysplastic lesions of the nasopharynx and invasive NPC combined can suggest that EBV may have the potential to drive neoplastic transformation of nasopharyngeal epithelial cells and facilitate the clonal expansion of malignant cells (Young and Dawson, 2014; Tsao et al., 2015).

#### Gastric Carcinoma (GC)

Recently various studies reported the presence of EBV in lympho-epigastric adenocarcinomas (Shibata et al., 1991). However, EBV contribution to pathogenesis in these tumors are still in question. Moreover, there are morphological similarities found between lympho-epithelioma-like gastric carcinoma (GC) and undifferentiated NPC (Young and Dawson, 2014). Hence, it has been hypothesized that in lympho-epithelioma-GC, EBV spreads from the nasopharynx to the stomach (Iizasa et al., 2012). While, in gastric adenocarcinomas EBV may perhaps enter the gastric epithelium without the use of a receptor (Thompson and Kurzrock, 2004). Recently, Chen et al. (2015) showed that all gastric mucosa samples from healthy populations were EBV RNA-negative, but interestingly EBV-positivity was found in 46% of patient tissues with gastritis. These patterns demonstrates that EBV infection may lead to induction of persistent gastric mucosa inflammation and subsequent carcinogenesis (Chen et al., 2015). Cancer Genome Atlas Research Network (2014), the cancer genome atlas research network characterized the genetics of EBV- vs. EBV+ GC, and interestingly found distinct mutations and epigenetic profiles in EBV+ GC cases. They demonstrated that EBV+ GC have recurrent PIK3CA mutations, high DNA hypermethylation, and also amplification of JAK2, CD274, and PDCD1LG2 (Cancer Genome Atlas Research, 2014).

## EBV Latent Infection of B-Cells

**FIGURE 1:**
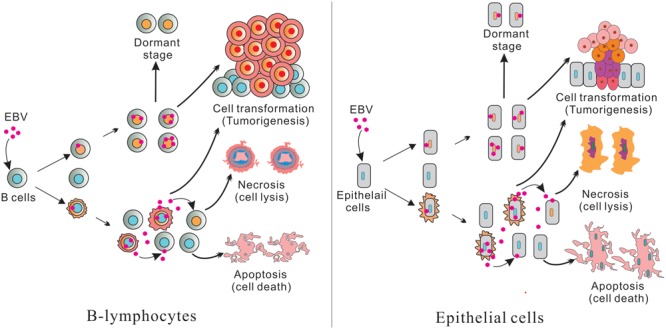
**(Left)** Represents B-lymphocytes infection and **(Right)** epithelial cell infection. Epstein–Barr virus (EBV) infected B-lymphocytes and epithelial cells have pools of uninfected and infected cells. Further, some cells produce infectious virus which can infect new cells. The remaining cells will die through apoptosis and necrosis. A portion of the infected cells are transformed and leads to tumorigenesis through cell transformation. Some infected cells are also switched to a dormant stage and can be activated or reactivated when conditions are favorable for lytic replication.

Epstein–Barr virus is perhaps best known for its ability to immortalize human primary B-lymphocytes in culture. A number of B-cell malignancies are associated with EBV infection, including eBL, classical Hodgkin lymphoma (cHL), diffuse large B-cell lymphoma (DLBCL), and AIDS-related lymphoma (Vockerodt et al., 2015). EBV is also found in NK/T-cell lymphoma and several epithelial malignancies like GC and NPC, and has potent B-cell transforming activity *in vitro* (Pope et al., 1968). Also, mimics B cell proliferative and survival signaling, which allows it to replicate its genome while remaining latent and immune-silent in the host B-cells, thus establishing lifelong persistence (Young and Rickinson, 2004; Cesarman and Mesri, 2007). Most EBV infection is asymptomatic, but EBV manipulation of host cell systems for latent persistence can lead to oncogenesis. Primary infection is usually asymptomatic or causes infectious mononucleosis (IM; **Figure 1**; Henle et al., 1968). After primary infection, EBV resides mainly in the long-lived memory B cells of infected individuals (**Figure 1**), but how EBV gets there is still a major unanswered question.

Different transcription programs are established in different tissues which maintains lifelong EBV infection. Since similar transcriptional programs are found in EBV cancers, the pattern of latency which determines the EBV genes expressed is a key component of the puzzle to understanding the role of EBV antigens in inducing cancers (Kutok and Wang, 2006; Cesarman and Mesri, 2007). The B-cell malignancies express the established latent gene transcription patterns during EBV infection and the related EBV antigens which contribute to the oncogenic process. This is likely linked to the agrressivity of the cancer, the response and recurrance of the malignancies suggesting that these viral antigens are important in driving these viral associated cancers.

Latency I (Lat I) program is characterized by the expression of EBNA1, EBERs, BARTs, and BART microRNAs (Marquitz et al., 2014). EBNA1, the only expressed viral protein in latent infection, tags the EBV episome to the host chromosome, thus allowing it to be segregated and retained during cell division (Frappier, 2012b). Furthermore, EBNA1 is essential for lymphoma survival by preventing cell death (Kirchmaier and Sugden, 1997). Characteristically, in Burkitt’s lymphoma (BL), none of the growth-promoting latent genes are expressed except the latent protein EBNA1 (Gregory et al., 1990). Although, the involvement of EBV in BL is supported by high frequency of tumors that carry the virus in endemic areas (98%), and the presence of clonal EBV in all the tumor cells (de-The, 1985; Gulley et al., 1992), it is still not completely understood how EBNA1 participates in tumorigenesis, a multistep process that occurs over a long period of time.

Latency II (Lat II) is characteristic of NPC, HD, Reed-Sternberg (RS) tumor cells, and EBV-infected T and NK lymphoma cells (Mesri et al., 2014). In addition to EBNA1, LMP1, and LMP2A are also viral antigens expressed in this latency program (Mesri et al., 2014). These two EBV oncogenes mimic key survival and proliferative signals in B-cells (Mosialos et al., 1995). To be specific, LMP1 mimics a constitutively active CD40 receptor (Mosialos et al., 1995), while LMP2A is able to mimic signaling via activated B cell Ig receptors depending on its tyrosine-based activation motif (ITAM; Merchant et al., 2000). The first recognition of an association between EBV and Hodgkin’s disease came from the observation that IM is a risk factor for this form of lymphoma (Flavell and Murray, 2000). Subsequently, immunoglobulin mutations and the data on viral gene expression independently support the idea that Hodgkin’s disease arises from an EBV-infected germinal-center B cell (Kuppers and Rajewsky, 1998).

The associated interaction between EBV and epithelial cells is poorly understood compared to B-cells. It has been shown that infection and replication of the virus usually happens in epithelial cells prior to infection of B cells (Shannon-Lowe et al., 2009). Recently, EBV replication was shown in tongue epithelium, hypothesizing that tongue could be the source of EBV and secreted into saliva (Herrmann et al., 2002). Although, OHL is well-known for productive EBV replication in upper-layer epithelial cells which undergoing terminal differentiation, apoptosis, and desquamation (Walling et al., 2001). All these activities clearly demonstrated that EBV replication *in vivo* is derived upon the differentiation state of the epithelial cells (Pegtel et al., 2004). Moreover, proper latency in EBV infected epithelial cells is mainly described in NPC and GC (Iizasa et al., 2012). NPC has been reported to be the expression of EBERs, BARTs RNA, EBNA1, LMP2, and LMP1 (Iizasa et al., 2012).

Latency III (Lat III) or growth program is characteristic of EBV-infected B-lymphocytes proliferating as long-term lymphoblastoid cell lines (LCLs) in cell culture. In this pattern, EBV expresses six nuclear antigen proteins (EBNA1, 2, 3A, 3B, 3C, and LP), two latent infection integral membrane proteins (LMP1 and LMP2A, 2B) in addition to the BARTs, EBERs, and miRNAs (Middeldorp et al., 2003; Thompson and Kurzrock, 2004; Bajaj et al., 2007; Hislop et al., 2007; Saha and Robertson, 2011). The expressed latent proteins play important roles by regulating cell cycle, cell proliferation and contribute to the oncogenic process and both latency II and III patterns drive oncogenesis in B- and epithelial cells (Cesarman and Mesri, 2007). Lymphomas expressing EBV latency III program typically develop in immunodeficient individuals including AIDS and transplant patients, while EBV lymphomas and other cancers in immunocompetent hosts will typically display latency I or II patterns (Kutok and Wang, 2006; Cesarman and Mesri, 2007).

A Latency 0 (Lat 0) pattern has also been described where only the EBV-encoded ncRNAs which include EBERs and BARTs are transcribed, and no viral proteins are expressed (Thorley-Lawson, 2001; Shaknovich et al., 2006). The infection status was observed in resting memory B cells. Therefore, the lack of EBV protein expression helps these cells to evade T cells recognition, but it is still unknown how viral proteins are regulated in this pattern, and what kind of cellular transcription factors are involved in the truly latent cells.

## EBV Nuclear Antigens and Their Contribution to Oncogenesis

### EBNA1

Epstein–Barr virus nuclear antigen 1 (EBNA1) was the first EBV protein detected and is expressed during both EBV latent and lytic infection (Reedman and Klein, 1973). EBNA1 is the only protein required for the persistence of EBV genomes through contribution to both the replication and mitotic segregation of the viral genome (Frappier, 2012b). Additional evidence suggests that EBNA1 is involved in regulation of viral and cellular gene expression (Pfeffer et al., 2004). For example, it is essential for lymphoma survival by preventing cell death (Kirchmaier and Sugden, 1997). EBNA1 induces survivin protein expression and activates its transcription activity by its Sp1 site at the promoter. The up-regulation of survivin expression will suppress cell apoptosis by inhibiting the caspase pathways in EBV-positive cells (Lu et al., 2011). EBNA1 can also induce genomic instability, including DNA damage response (DDR), chromosomal aberrations and DNA double-strand breaks (DSBs), by regulating RAG-1 and RAG-2 and increasing reactive oxygen species (ROS) through activating the transcription activity of NOX2/gp91^phox^, a catalytic subunit of NADPH oxidase (Tsimbouri et al., 2002; Gruhne et al., 2009; Maynard et al., 2009). A recent study shows that phosphorylation of EBNA1 serine 383 by ERK2 is crucial for EBNA1-mediated transactivation (Noh et al., 2016). In addition, EBNA1 inhibits the expression of the protein tyrosine phosphatase receptor kappa (*PTPRK*), a TGF-β target gene, and helps the survival and growth of HD cells (Flavell et al., 2008). In NPC, EBNA1 could regulate cell metastasis and migration by increasing the expression and nuclear localization of Nm23-H1 which is involved in metastases (Murakami et al., 2005; Cao et al., 2012). EBNA1 also activates transcription activity of AP-1, and further enhances the expression of its target protein vascular endothelial growth factor (VEGF) in NPC cells, which suggests EBNA1 may contribute to angiogenesis and metastasis of NPC (O’Neil et al., 2008). EBNA1 can disturb PML nuclear bodies and then inhibit malignant transformation, which may be important for the development of NPC (Sivachandran et al., 2008). Therefore, a large body of evidence has shown that EBNA1 is associated with several types of cancer through dysregulation of multiple signaling pathways.

### EBNA2

Epstein–Barr virus nuclear antigen 2 (EBNA2) is one of the initial latent viral genes expressed during EBV infection. EBNA2 initiates the transcription of a cascade of primary and secondary target genes through activation of several viral and cellular genes (Maier et al., 2006). These changes eventually regulates the activation of the resting B cell, through the cell cycle inducing proliferation of growth transformed cells (Spender et al., 1999). EBNA2 is a nuclear phosphoprotein that mimics intracellular cleaved Notch1 and associates with RBP-Jk to activate the expression of Notch1 target genes (Hofelmayr et al., 2001), and deregulated Notch signaling is known to drive non-viral lymphoid malignancies (Kaiser et al., 1999). EBNA2 is also able to deregulate expression of c-MYC, further increasing cell proliferation by up regulating cyclin Ds and E, as well as downregulation of CDK2 inhibitors such as p21CIP1 and p27KIP1 (Kaiser et al., 1999). EBNA2 also down-regulates the expression of Bcl6, a master regulator of germinal center differention, suggesting a key role of EBNA2 in B cell lymphomagenesis (Boccellato et al., 2007). A recent study showed that the overall survival of patients with EBNA2-positive DLBCL was dramatically poorer than patients with EBNA2-negative DLBCL, suggesting an important role for EBNA2 in lymphoma development (Stuhlmann-Laeisz et al., 2016).

### The EBNA3 Family of Proteins

Epstein–Barr virus nuclear antigen 3 (EBNA3) is a family of three latency-associated proteins, which includes EBNA3A, EBNA3B, and EBNA3C. They were first identified in latently infected B cell cultures, and appear to be critical for EBV persistence and B-cell lymphomagenesis (Whitehurst et al., 2015). The EBNA3 family of proteins share approximately 30% sequence homology, and are all expressed during the latent phase of EBV infection of primary B-cells and in EBV-associated tumors of immunocompromised cells. However, only EBNA3A and EBNA3C are essential for viral transformation of B-lymphocytes, and all appear to significantly contribute to maintaining the viability of transformed cells, suggesting an important role in oncogenesis (Tomkinson et al., 1993). EBNA3C has been reported to interact with many cellular factors (Robertson et al., 1995; Choudhuri et al., 2007; Saha et al., 2011, 2015; Banerjee et al., 2013, 2014; Jha et al., 2013a, 2014, 2015a,b,c). One of these cellular antigens is RBP-JK, which binds to all the EBNA3 proteins (Robertson et al., 1995, 1996). EBNA3C also binds with Nm23-H1 and reverses its ability to induce cell migration (Subramanian et al., 2001), and targets cell-cycle checkpoints by engaging the SCF (Skp2, Cullin, F-Box)-ubiquitin ligase complex that can also lead to proteasomal degradation of the retinoblastoma tumor suppressor protein which demonstrates one of the mechanisms of oncogenesis induced by EBV through the EBNA3 family of proteins (Knight et al., 2005).

**FIGURE 2:**
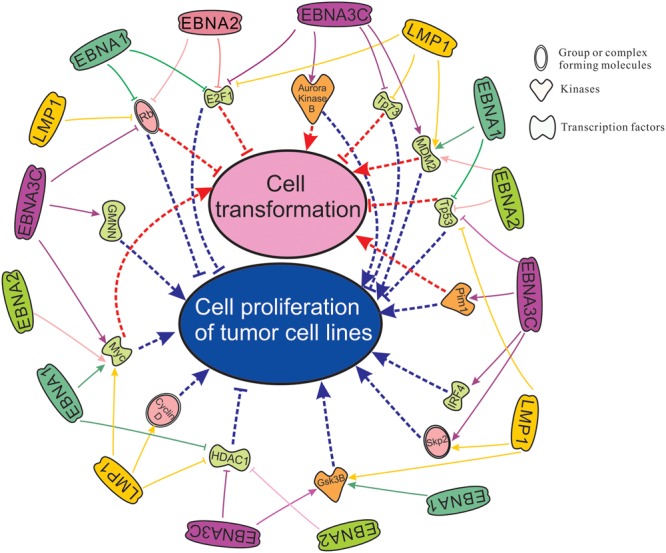
**Cell transformation and cell proliferation of tumor cell lines as mapped using the IPA software which demonstrates significant association with major cellular pathways.** Here we have groups or complexes formed with cellular molecules including Rb, Skp2, CyclinD, the transcription factors E2F1, Tp73, Mdm2, Tp53, IRF4, Myc, HDAC1 and GMNN (Geminin), and kinases which include Aurora Kinase B, Pim1 and Gsk3B. We have also mapped the known regulation of these cellular antigens and their activities by the essential EBV latent antigens EBNA1, EBNA2, EBNA3C, and LMP1 for transformation and immortalization of EBV-infected cells leading to associated cancers.

There are two important phenomenon in the process of EBV induced oncogenesis described through several cellular players in this review. We have utilized Ingenuity Pathway Analysis (IPA) software, a product of Qiagen, Redwood City, CA, USA. The available database included cellular players that are significantly associated (*p*-value is ∼10^-13^) with transformation and proliferation of tumor cell lines (*p*-value is ∼10^-12^) (**Figure 2**). Our lab and others have demonstrated that EBNA1, EBNA2, EBNA3C, and LMP1 are potent EBV antigens associated with a number of transcription factors including E2F1, Tp73, MDM2, Tp53, IRF4, Myc, HDAC1, and GMNN (Robertson et al., 1995, 1996; Kaiser et al., 1999; Ma et al., 2000; Plaxco et al., 2000; Zhou et al., 2000, 2005; Knight et al., 2005; Liu et al., 2005; Saridakis et al., 2005; Choudhuri et al., 2007; Ding et al., 2007; Chau et al., 2008; Allday, 2009; Forte and Luftig, 2009; Borestrom et al., 2012; Frappier, 2012a; Accardi et al., 2013; Banerjee et al., 2013; Tursiella et al., 2014; Jha et al., 2015b,c; Saha et al., 2015), kinases including Aurora Kinase B, Pim1, GSK3B (Saha et al., 2011; Jha et al., 2013a; Banerjee et al., 2014; Edwards et al., 2015), and forms complexes with cellular factors whcih includes the tumor suppressor and oncoproteins, Rb, Skp2, and CyclinD (Arvanitakis et al., 1995; Ruf and Sample, 1999; Prathapam et al., 2002; Knight et al., 2005; Choudhuri et al., 2007; Kang et al., 2011; Saha and Robertson, 2011; Saha et al., 2011, 2015; Sun et al., 2015; **Figure 2**). Cell transformation is regulated through a broad range of interacting partners including Rb, E2F1, Aurora Kinase B, Tp73, Mdm2, Pim1, Tp53, IRF4, Skp2, and Myc (Halazonetis and Kandil, 1991; Lahoz et al., 1994; Hoang et al., 1995; Pirollo et al., 1997; De Laurenzi et al., 1998; Givol et al., 1998; Mochizuki et al., 1999; Lin et al., 2000; Gstaiger et al., 2001; Kanda et al., 2005; Zhang et al., 2005; Xu et al., 2011). Moreover, proliferation of tumor cell lines is associated with dyregulation of the activities of the major cell factors Rb, E2F1, Aurora Kinase B, Tp73, Mdm2, Pim1, Tp53, IRF4, Skp2, Myc, HDAC1, Cyclin D, GSK3B, and GMNN (Wang et al., 2008; Liontos et al., 2009; Karslioglu et al., 2011; Inuzuka et al., 2012; Jo and Ren, 2012; Maddocks et al., 2013; Malinen et al., 2013; Huo et al., 2014; Mikawa et al., 2014; Subramanian et al., 2015). These studies together highlight the importance of EBNA3C and other EBNA proteins in EBV-directed oncogenesis.

### The LMP Family of Proteins

Latent membrane protein-1 (LMP-1) mimics CD40 and its over-expression leads to activation of NF-kB, JNK, PI3K/Akt, and MAPK pathways to promote cellular proliferation (Shair et al., 2007). Additionally, LMP1 activated Bcl2 blocks apoptosis and participated in cell cycle progression through cyclinD/CDK2 phosphorylation of Rb, and inhibition of p16 and p27 (Everly et al., 2004). Another member of the LMP family, LMP2A diminishes the surface immunoglobulin-mediated lytic cycle reactivation (Bryant and Farrell, 2002). LMP2A also induces the Ras/PI3K/Akt pathway and activates Bcl-xL expression to promote B-cell survival (Chen, 2012). A study by Accardi et al. (2013), showed that LMP1 activates the expression of ΔNp73α, a strong antagonist of p53. Furthermore, inhibition of JNK1 through chemicals or silencing, strongly down-regulated ΔNp73α mRNA levels in LMP1 positive cells (Accardi et al., 2013). At the same time, LMP1 mutants deficient in induction of JNK1 did not induceΔNp73α accumulation (Accardi et al., 2013). Interestingly, LMP1 also induces epigenetic changes on the promoter of p73 gene (Accardi et al., 2013). Emerging studies suggest that these epigenetic changes play an important role in carcinogenesis (Ren et al., 2011). Additionally, knockdown through short hairpin RNA depletion of endogenous LMP1 in LCLs suppressed IL-32 expression, a novel proinflammatory cytokine (Lai et al., 2015). Moreover, IL-32 can bind with protein kinase Cδ inhibiting activation of the Zta promoter. Therefore, knockdown of IL-32 in LCLs induces viral reactivation. However, and surprisingly this has no influence on cell proliferation and apoptosis (Lai et al., 2015).

### Non-coding RNAs

The EBV-encoded nuclear BART RNAs are functional long non-coding RNAs (lncRNAs) (Marquitz et al., 2015). To avoid host immune attack, EBV expression of non-coding RNAs that contribute to growth regulation without an immune response may have implications for immune escape by the virus (Marquitz et al., 2015). A recent RNA-seq analysis by Chen et al. (2005) had showed that more than 99% of all virally derived polyadenylated transcripts were BARTs in EBV-infected gastric tumors. Another study by Feederle et al. (2011) highlighted the functions of miRNAs in EBV infection as a strategy for allowing synchronous and synergistic expression of genetic elements that contribute to transformation of their target cells.

EBV-encoded small RNAs (EBERs) has been reported as growth-stimulatory and their roles have been confirmed in EBV-mediated B-cell transformation (Yajima et al., 2005). Using genetic engineering approaches by reverse genetics in primary B cells and in Akata cells the data strongly supports the hypothesis that EBERs have roles in transformation of human primary B cells. However, *in vitro* studies with deletion mutants have indicated that EBV can transform B-cells without the presence of the EBERs (Jha et al., 2016).

## The Switch of Lytic Infection and Latency

Typically, two types of EBV infection can be established in different cell types: latent in primary B-cells and lytic in epithelial cells (**Figure 1**; Amon and Farrell, 2005; Tsurumi et al., 2005). Primary infection is generally asymptomatic. However, primary infection in early adulthood can lead to IM (Odumade et al., 2011). Upon initial infection of cells, EBV briefly undergoes an initial burst of lytic replication that is productive but is aborted and switches to a latent infection (**Figure 1**; Mansouri et al., 2014). Following this period, EBV becomes strictly latent after the lytic genes are silenced by chromatinization (Penkert and Kalejta, 2011). It is important to note that only a small fraction of the virus infected cells are reactivated during the latent infection state (**Figure 1**; Rong and Perelson, 2009).

The switch during EBV infection in host cells from latent to lytic form is greatly determined by the cellular transcription factors that regulate the activity of immediate-early (IE) lytic genes, especially *BZLF1* and *BRLF1* (Adamson et al., 2000). In EBV latently infected cells, multiple cellular factors are necessary for establishment and maintenance of viral latency (Haan et al., 2001). For example, YY1 inhibits *BZLF1* and *BRLF1* transcription and is important for maintenance of viral latency (Zalani et al., 1997). MEF2D recruits class II histone deacetylases (HDAC) to the *BZLF1* gene promoter and inactivates its activity (Gruffat et al., 2002b). Furthermore, Oct-2 and PAX5, two B cell-specific transcription factors, have also been shown to be important in promoting EBV latency by negatively regulating the function of the BZLF1 protein (Robinson et al., 2012; Raver et al., 2013). The chromatin structure of the *BZLF1* gene promoter (Zp) is also critical for regulating its expression (Chang et al., 2008). Zp is activated by histone acetylation and was shown to be inactivated by DNA methylation (Bryant and Farrell, 2002). These multiple strategies are involved in regulating latency during EBV infection.

Epstein–Barr virus reactivation can be triggered in human cells by either chemical agents or biological stimuli, including TPA, sodium butyrate, HDAC inhibitors, phorbol esters, calcium ionophores, chemotherapeutic agents, BCR engagement, TGF-β, and hypoxia (**Figure 1**; Gorres et al., 2014; Kenney and Mertz, 2014). BCR signaling-mediated EBV reactivation is mainly induced through PI3K and PKC that further regulate the activity of the Zp promoter (Goswami et al., 2012). T-cells secreting TGF-β also triggers EBV reactivation through signaling and induction of *BZLF1* gene expression (Fahmi et al., 2000; Inman and Allday, 2000). Hypoxia can also activate EBV lytic infection by enhancing the expression of the immediate early transactivator Zta (Jiang et al., 2006). These factors finally activate the transcription of two lytic transactivators *BZLF1* and *BRLF1* (Miller et al., 2007). Then the two genes encode antigens induced expression of viral E (early) genes, which are necessary for viral DNA replication, and L (late) genes, such as capsid proteins and glycoproteins (Gruffat et al., 2002a). In most EBV-positive cell lines, the Zta protein, encoded by the *BZLF1 ORF*, is a major trigger for viral reactivation because its expression alone can induce the reactivation cascade (Speck et al., 1997). This suggests that it is a major switch important for EBV reactivation from latency. Expression of the *BRLF1* gene encoding the Rta protein also induces the switch from latent to lytic infection in a subset of EBV-positive cells, particularly epithelial cells. Importantly, EBV reactivation is largely induced by BCR stimulation and plasma cell differentiation (Laichalk and Thorley-Lawson, 2005; Davies et al., 2010). Two cellular proteins, XBP-1 and BLIMP-1, are critical for differentiation of plasma cells (Reimold et al., 2001; Shaffer et al., 2002). Furthermore, the expression of XBP-1 in some EBV latently infected cells is sufficient for viral reactivation, and BLIMP-1 could also induce viral lytic cycle in EBV-transformed cells by activating Zp and Rp transcription (Vrzalikova et al., 2011).

## Reactivation of EBV

Viral reactivation in an infected host is induced by several key factors (Ye et al., 2011). Chronic interpersonal stress can drive EBV reactivation and replication by weakening the cellular immune system control over viral latency through both autonomic and glucocorticoid pathways (Fagundes et al., 2014). The chronic relationship with stress could be a key psychological feature driving the link between attachment anxiety and EBV reactivation (Fagundes et al., 2014). Also, it has been hypothesized that individuals who have higher attachment anxiety have elevated EBV VCA IgG antibodies compared to lower attachment anxiety (Fagundes et al., 2014).

Typically, EBV-induced cancers containing undifferentiated cells may be due in part to cells dying from lytic EBV infection when they differentiate (Reusch et al., 2015). However, Ntera-2 cells, a neuro-epithelial cells can be infected by EBV after differentiation (Jha et al., 2015a). In gastric cancer and NPC cell lines, BLIMP1 is sufficient to induce EBV lytic gene expression (Reusch et al., 2015). Furthermore, the studies showed that BLIMP1 can activate transcription of Rp over 300-fold in addition to Zp at 20- to 50-fold in several epithelial cell lines (Reusch et al., 2015). Reusch et al. (2015) showed that both Zp and Rp are robustly induced by BLIMP1, and that Rp is activated greater than Zp in epithelial cells. Therefore, it is possible that BLIMP1 activation of Zp is a major mechanism for induction of EBV reactivation in B-cells (Vrzalikova et al., 2011). Therefore, it is speculated that EBV induced B-cell malignancy are much more responsive to Zp than Rp (Reusch et al., 2015). Also, deletion of EBER1 or EBER2 had minimal effect on the transformation frequency of primary B-cells or the generations of LCLs by EBV (Wu et al., 2007). Moreover, Gulley and Tang (2008) showed that EBER2 is essential for efficient transformation of B lymphocytes and maximum growth potential of LCLs. Contrary to this, several studies demonstrated that EBERs were not essential for primary infection, viral replication, or B-cell immortalization (Delecluse and Hammerschmidt, 2000). However, EBERs were found to be important for establishment of malignant phenotypes and tumor formation in SCID mice (Yamamoto et al., 2000).

Most of the EBV latent proteins expressed in Wp-restricted or type III latency were oncogenic and might contribute to resistance of EBV-associated lymphomas to chemotherapy (Hui et al., 2014). Further, it was shown that EBV-positive BL cells of type III latency were more resistant to dying by nocodazole or taxol compared to EBV negative or latency I BL cells (Leao et al., 2007). BL cells of Wp-restricted or type III latency were more resistant to treatment with HDAC inhibitors than those of type I latency (Leao et al., 2007).

TERT-induced NOTCH2 activation is regulated through NF-kB (Nickoloff et al., 2002). In addition, pharmacologic inhibition of NOTCH signaling triggers EBV lytic cycle which leads to the death of the EBV-infected cells (Giunco et al., 2015). IRFs are also regulated by EBV and can modulate the expression of both viral and cellular factors that are involved in EBV latency and transformation (Zhang and Pagano, 2001). To date, it is known that IRF-7, IRF-4 and IRF-5 are associated with EBV transformation (Xu et al., 2011). IRF-5, normally a tumor suppressor is highly expressed in EBV transformed cells. Combined with IRF-4, it is involved in EBV-mediated regulation of Toll like receptor 7 (TLR7) activities (Martin et al., 2007). Recently, we have shown that Spi-1/B motif of IRF-4 is critical for its interaction with EBNA3C in EBV-induced B-cell immortalization (Banerjee et al., 2013). We also demonstrated that EBNA3C can stabilize IRF-4, which leads to downregulation of IRF-8 by enhancing its proteasome-mediated degradation (Banerjee et al., 2013). Therefore, we conclude that EBV induces a balanced expression of IRFs during EBV transformation which when deregulated can trigger reactivation. With mutual inhibition and/or activation among oncogenic and tumor suppressor factors, EBV may drive the infected cell to apoptosis or proliferation in various microenvironments for the survival of the virus *in vivo* and this may also have important consequences for viral gene regulation whcih leads to reactivation of a small percentage of infected cells in tumors which are critical for maintaining the cancer phenotype (Zhao et al., 2010).

In LCLs and PBMCs, we have successfully demonstrated that the mitotic protein Aurora kinase B (AK-B) is critical, and regulated through EBNA3C in B-cell transformation (Jha et al., 2013a). In the course of transformation both EBNA3C and AK-B targets several tumor suppressors like p53, p73 and pRB through a mechanism of phosphorylation and ubiquitination (Jha et al., 2015c). Cyclins, specifically Cyclin A, D1, E are also a crucial regulators in B-cell transformation through EBV (Saha et al., 2011). Several studies highlighted the importance of the DDR in EBV induced B-cell transformation (Choudhuri et al., 2007; Jha et al., 2013b, 2014). Briefly, ChK1, ChK2, and H2AX are critical components of EBV-derived B-cell transformation and are activated during early infection as well as provide a bridge to bypass the host immune system for virus propagation (Choudhuri et al., 2007; Jha et al., 2014).

Studying latent and lytic replication in EBV infected B-cells or epithelial cells is important for determination of the mechanisms which lead to development of EBV-associated diseases. In several studies utilizing primary or immortalized B-cells infected with wild type EBV isolated from BAC clones, we monitored the expression of genes at transcript levels during the early days of infection (**Figure 1**; Halder et al., 2009; Saha et al., 2011, 2015; Banerjee et al., 2013; Jha et al., 2013a, 2015b,c). Further, we have used several epithelial primary and immortalized cells including primary neurons, Ntera-2, Sh-Sy5Y, and HEK-293 cells (Jha et al., 2015a), and clearly showed that during the initial days of EBV infection the latent gene expression was low, but the lytic gene expression resulted in active virus progeny produced in the culture medium. Furthermore, this active virus progeny was capable of infecting new cells during this early infection period. Therefore, the number of EBV infected cells were greater on day 5 compared to days 1 or 2 (Jha et al., 2015a). Active virus progeny were verified through infection of fresh cells with virus collected from media during early infection (**Figure 1**). We have also observed that a large percentage of infected cells died during this early period. Previous studies have also suggested that EBV-infected B-cells are transformed to LCLs in a similar manner after and initial period of lytic virus production (**Figure 1**; Kurth et al., 2000; Carter et al., 2002; Shannon-Lowe et al., 2009). However, investigating the disease-associated cell types will provide further insights which will enhance our understanding of the complexity of EBV-driven pathogenesis in a range of associated chronic diseases.

## EBV Viral Load in Hodgkin and Non-Hodgkin

Epstein–Barr virus viral load in plasma has been used as a marker of tumor burden in patients with sporadic EBV-related lymphoma including B cell, T cell, NK cell, and Hodgkin subtypes (Gulley and Tang, 2010). Interestingly, EBV DNA is detectable ahead of cancer diagnosis, and also the level of its DNA at diagnosis can predict effects and efficacies of therapies (Zhang et al., 2015). These findings strongly suggest that routine plasma evaluation for EBV viral load on initial diagnosis may be important for predicting EBV-related pathogenesis. Whether or not this is transferable to other know tumor viruses is yet to be determined but in this age of microbiome and exchange of genetic information between host and infectious agents it would not be surprising if future diagnostic strategies can be developed using blood serum analysis of nucleic acids signatures to detect levels of infectious agents including viruses like EBV as a routine test for predicting associated pathologies.

## Treatment of EBV Associated Diseases

To date the majority of antiviral agents used against EBV are the acyclic nucleoside analogs Acyclovir or Ganciclovir, which are only efficient during EBV lytic life cycle (Gustafson et al., 1998). Another antiviral Maribavir is active against EBV (Gershburg and Pagano, 2005). However, the mechanism of action is not fully understood. Furthermore, some natural and synthetic compounds, like moronic acid, derivatives of betulinic, glycyrrhizic acids, and flavonoids have been shown to inhibit EBV lytic cycle (Chang et al., 2003; Lin, 2003; Lin et al., 2008). However, their mechanism of actions are not well-defined.

Recently, we have successfully identified several small molecules inhibitors (NSC65381, NSC10010, NSC16553, and NSC1881) that were able to kill virus positive LCLs as well as EBV negative BL cells (Dzeng et al., 2015). We have also demonstrated that c-Myc and NF-kB are the major signaling molecules targeted through these inhibitors to kill virus positive cells (Dzeng et al., 2015). Earlier, arsenic trioxide (As2O3) and sodium arsenite (NaAsO2) were also shown to induce cell death in P3HR1 cells (Zebboudj et al., 2014). Interestingly, this effect was due to reactivation of EBV lytic cycle through induction of the immediate-early proteins Zta and Rta (Ragoczy et al., 1998). There are several studies using a combination of drugs including rituximab, cyclophosphamide, vincristine, doxorubicin, and prednisolone or methotrexate, vincristine, and procarbazine to treat EBV associated patients with greater efficacy (Kuriyama et al., 2014). These combined therapeutic approaches may be a better strategy for future anti-viral therapy as is now becoming the norm in most oncology regimens.

## Potential Future Discovery

Recently growing evidence suggest that the CRISPER/CAS9 system can be efficiently activated in viral infected cells for editing of viral genomes (Seeger and Sohn, 2014; Yuen et al., 2015). Using this strategy we can precisely destroy the latent promoters of EBV which are critical for viral latent infection and so result in loss of transformed phenotype and killing of infected cells. This could be a potential strategy for control of EBV pathogenesis in immunocompromised individuals. Another approach for future exploration is to uncover the complete transcription network of host-EBV interaction. This could be classified as the types of genes at different locations, temporally expressed, and the associated pathologies. One interesting possibility is the utility of EBV in gene therapy. EBV can efficiently infect B-cell and the infected cells circulates in the vascular system. Therefore, EBV may have potential for use as a novel vector to transport of cellular genes to specific anatomical sites as a means of targeted gene therapy.

## Author Contributions

All authors listed, have made substantial, direct and intellectual contribution to the work, and approved it for publication.

## Conflict of Interest Statement

The authors declare that the research was conducted in the absence of any commercial or financial relationships that could be construed as a potential conflict of interest.
